# Integrative Analysis of Novel Ferroptosis‐Related Genes Signatures as Prognostic Biomarkers in Ovarian Cancer

**DOI:** 10.1002/cnr2.70284

**Published:** 2025-07-31

**Authors:** Leilei Cao, Yiqin Ouyang, Wei Lu, Xiao Qi, Zhijie Wang, Jingshuai Wang

**Affiliations:** ^1^ Department of Obstetrics and Gynecology Shanghai Eighth People's Hospital Shanghai China; ^2^ Department of Obstetrics and Gynecology Shanghai East Hospital, Tongji University School of Medicine Shanghai China

**Keywords:** ferroptosis, immunotherapy, ovarian cancer, tumor microenvironment, tumor mutation burden

## Abstract

**Background:**

Ferroptosis, an iron‐dependent form of cell death, has been implicated in the pathogenesis of several types of cancer. Nevertheless, the exact correlation between ferroptosis‐related gene mutations and their influence on ovarian cancer (OV) diagnosis and treatment strategies remains to be fully elucidated. It is crucial to identify the ferroptosis‐related gene signature in OV and elucidate the impact of these mutations and their expression on the diagnosis and treatment of OV.

**Methods:**

In this study, we collected data from the TCGA and GEO databases. We utilized various tools and packages for data analysis, including the cBio Cancer Genomics Portal, Tumor Immune Estimation Resource (TIMER), GSVA package, and WGCNA R packages.

**Results:**

Our results showed that ferroptosis subtypes 1 (FS1) and 2 (FS2) exhibited different levels of expression and tumor mutation burden (TMB). FS2 had a higher TMB level and survival rate compared to FS1. Furthermore, our analysis identified three ferroptosis‐related genes, including IFNG, KEAP1, and PHKG2, as key biomarkers in prognosis prediction and potential targets for OV cancer therapy. The elevated expression levels of IFNG, KEAP1, and PHKG2 were found to be correlated with a good prognosis. These three genes showed a positive correlation with TMB in OV. We also observed that high TMB was more robustly associated with immune response‐related gene expression, including CD28, CD40L, and type I IFN family members. Moreover, high TMB was associated with increased T cell infiltration and exhibited a distinct gene signature, which highlights the potential of IFNG, KEAP1, and PHKG2 as predictive markers for T cell infiltration and the tumor microenvironment status in OV. A significant correlation exists between the expression levels of KEAP1 and PHKG2 and TMB in OV cell lines.

**Conclusion:**

In conclusion, our study identified KEAP1, IFNG, and PHKG2 as potential prognostic biomarkers and therapeutic targets in OV. Their expression and mutation burden were correlated with a good prognosis. The association between ferroptosis subtypes, TMB, and survival rates further supports the relevance of these biomarkers. Additionally, the positive correlation between KEAP1, IFNG, and PHKG2 with TMB and immune response‐related gene expression highlights their potential as predictive markers for immunotherapy efficacy in OV. The observed association of high TMB with increased T cell infiltration and distinct gene signature further emphasizes its role as a potential biomarker for immune response. Further research is warranted to validate these findings and explore their clinical implications in OV treatment.

## 1 Introduction

Ovarian cancer (OV) is one of the most common women's cancers worldwide, and the majority of OV patients suffer relapse [1]. Indeed, within the realm of cellular biology, various types of cell death have been identified, including apoptosis, autophagic cell death, necrosis, pyroptosis, and ferroptosis [2]. Ferroptosis was first reported by Erastin in 2012. Ferroptosis was correlated with many diseases' progression and therapy. Studies reported that ferroptosis was mainly induced by iron metabolism disorders and was associated with drug resistance [3]. Iron plays an important role in ferroptosis, and ROS generation is the main feature [4].

The lipid metabolism has been reported to participate in the process of ferroptosis. The unique cell death mechanism of ferroptosis has aroused great interest in clinicians and scientists [5]. The role of ferroptosis in cancer progression and cancer therapy has been reported. The ferroptosis resistance in immune cells changed cell function and promoted breast cancer and colorectal cancer metastasis [6, 7]. The inhibition of ferroptosis by GST1 promoted gastric cancer metastasis [8]. Cancer cells resistant to drugs are sensitive to ferroptosis‐related therapy [9].

Recent researches indicate that certain cancer cells, such as mesenchymal and dedifferentiated cancer cells, exhibit heightened sensitivity to ferroptosis. Ferroptosis is recognized as an attractive target for cancer therapy, especially for refractory tumors. Moreover, the modulation of ferroptosis pathways has shown promise in overcoming resistance to conventional therapies, making it a critical area of exploration for improving patient outcomes [10, 11, 12]. Several studies also have focused on the regulatory networks of ferroptosis and the mechanisms of ferroptosis‐related genes (FRGs) in OV cancer [13, 14, 15, 16]. Targeting ferroptosis in cancer therapy is critically needed. Identifying the correlation of ferroptosis subtypes and the clinical relevance lies in its potential to revolutionize cancer treatment by enabling more precise targeting and personalization of therapies, ultimately improving patient outcomes. Despite the rapid growth in ferroptosis research, we know little about the correlation of ferroptosis subtypes with tumor mutation burden (TMB) and immune regulation.

In this investigation, data were gathered from the TCGA and GEO databases, following which a meticulous analysis was conducted utilizing a combination of tools and resources. The cBio Cancer Genomics Portal, Tumor Immune Estimation Resource (TIMER), as well as R packages including the GSVA package and WGCNA R package, were instrumental in the data analysis process. The statistical analysis was performed using the R package. We identified 2 ferroptosis subtypes (FS1 and FS2) in OV. The identified IFNG, KEAP1, and PHKG2 are positive with good prognosis. The TMB and tumor‐infiltrated immune cells are correlated with FS1 and FS2. The level of TMB in FS2 was higher than that in FS1. These findings suggest that targeting different ferroptosis subtypes is necessary when considering ferroptosis‐based treatments for OV. Simultaneously targeting ferroptosis, mutations, and immune checkpoints may yield better outcomes in clinical OV cancer therapy.

## 2 Materials and Methods

### 2.1 Data Collection and Preprocessing

**FIGURE 1:**
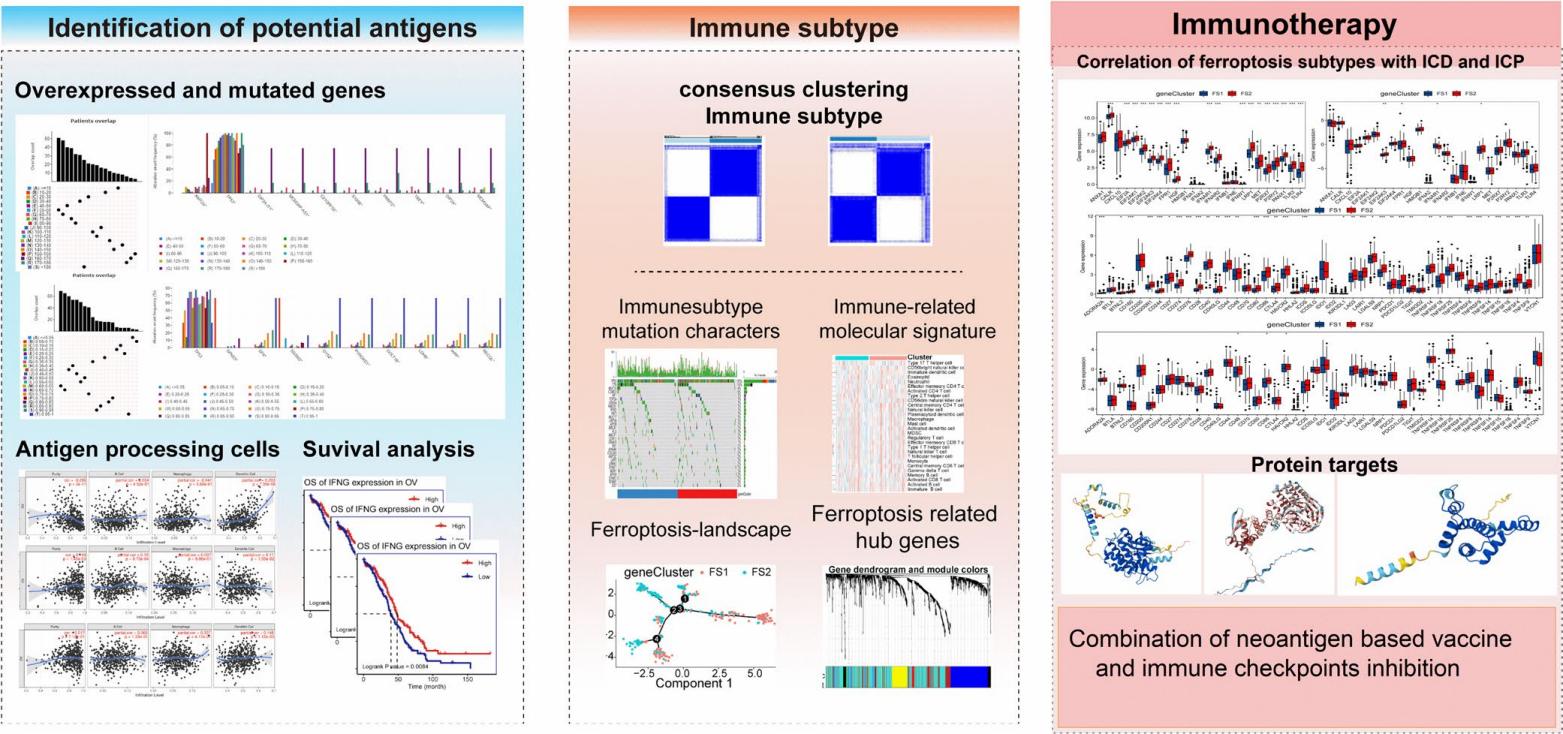
The flow chart and possible mechanisms of the analysis.

The data were collected from GDC (Genomic Data Commons) and the UCSC Xena database (https://xenabrowser.net/datapages/). Both TCGA OV samples (*n* = 379) and GTEx normal samples (*n* = 88) were used for further analysis. The FPKM of counting reads in features with HTSeq‐Counts and HTSeq‐FPKM was converted to TPM in R. The somatic mutation data of OV samples were selected using the VarScan and maftools R package. The relevant clinical information of patients was downloaded and analyzed. A total of 354 samples were selected for further analysis. The GSE32062 dataset from GEO was downloaded and 260 samples containing survival time were included in this study [17]. A total of 424 FRGs were acquired from the FerrDB database, enriching the pool of genetic information pertinent to the study of ferroptosis and its implications in various biological contexts [18]. The detailed methodology involves utilizing the GDC and UCSC databases to conduct an intersection analysis of differentially expressed genes associated with OV and genes related to ferroptosis. The identified genes from this intersection will subsequently undergo further validation using the GEO dataset (GSE32062). The immunogenic death‐related genes and the immune checkpoint genes were collected from previously published literature [19, 20]. The workflow diagram for this study was shown in Figure 1.

### 2.2 Analysis of the Correlation Between Genomic Variations and Prognosis

The multidimensional cancer genomics data of OV patients were obtained from the cBio Cancer Genomics Portal (http://www.cbioportal.org) [21]. The mutation count and fraction of the genome altered were analyzed. The microsatellite instability (MSI) and TMB were extracted using cBio Cancer Genomics Portal. In our study, we employed the DESeq2 package for analyzing the differential expression between OV patients and a healthy control group [22]. The absolute value of log_2_FC is greater than 1 and the *p* < 0.05 were considered as significantly altered in the context of our analysis. The Kaplan–Meier curve was used to describe survival characteristics. The log‐rank test was used to compare the survival times, and the *p*‐value less than 0.05 means statistically significant differences.

### 2.3 The Analysis of Factors Associated With Immune Cell Infiltration Using TIMER

Tumor Immune Estimation Resource (TIMER, https://cistrome.shinyapps.io/timer/) [23] was used to evaluate the factors affecting immune cell infiltration in the tumor microenvironment (TME). The correlation between the levels of immune cell infiltration and mutation burden was analyzed. The assessment of tumor cell purity within the Tumor Microenvironment (TME) was conducted using Spearman correlation analysis in our study.

### 2.4 Evaluation of the Correlation Between Ferroptosis Subtypes and Prognosis

FRG expression data were extracted from the expression matrix to construct a consistency matrix and identify the corresponding ferroptosis subtypes and gene modules through the “Consensus Cluster Plus” R package [24]. The K‐Medians Clustering and 1‐Pearson correlation with 1000 replications, resampling 70% of the patients in the cohort each time were used. The value of the cluster set ranges from 2 to 9, and the optimal partition is defined by evaluating the consensus matrix and consensus cumulative distribution function. Subsequently, the ferroptosis subtypes identified were validated in an independent GSE32062 cohort within the same experimental framework.

In our analysis, we utilized the log‐rank test to assess the prognostic significance of distinct ferroptosis subtypes. Furthermore, we employed Analysis of Variance (ANOVA) to investigate the correlation between ferroptosis subtypes and various ferroptosis‐related molecular characteristics, as suggested by Larson [25]. To evaluate immune enrichment scores, we applied the Single‐sample Gene Set Enrichment Analysis (ssGSEA) method from the GSVA package, enabling the calculation of immune enrichment scores associated with different ferroptosis subtypes in the context of OV [26].

### 2.5 Construction of Gene Co‐Expression Network and Ferroptosis Landscape in TME

Dimensionality reduction analysis based on graph learning and the dimensionality reduction function of the Monocle package with Gaussian distribution was used to visualize the distribution of ferroptosis subtypes in individual cancer patients. The DDRTree package was used to judge the dimensionality reduction method. Finally, the ferroptosis landscape was visualized using the color‐coding method. The gene expression, damaging mutations, hotspot mutations, and copy number variations of IFNG, KEAP1, and PHKG2 in different OV cell lines were analyzed using the Dependency Map (DepMap) portal website (https://depmap.org/portal/). mRNA was extracted from TOV‐112D and SKOV‐3 cell lines following the protocol of the RNeasy Universal Kit (Qiagen), and cDNA was synthesized using the cDNA Synthesis Kit (Qiagen). The gene expression levels were determined using the One‐Step qRT‐PCR Kit (TaKaRa, Dalian, China), and the reactions were conducted using the ABI7500 System (Applied Biosystems, CA, USA).

### 2.6 Statistical Analysis

In this study, all statistical analyses were conducted using R software (version 4.1.1). The Wilcoxon rank sum test was employed to assess differences between two groups, while the Kruskal–Wallis test was utilized to evaluate variances among more than two groups. Spearman correlation analysis was applied to explore associations between variables. A significance level of *p* < 0.05 was considered statistically significant, guiding the interpretation of the findings within the context of the study on ferroptosis‐related mutations in OV.

## 3 Results

### 3.1 Identification of Ferroptosis‐Related Mutation

**FIGURE 2:**
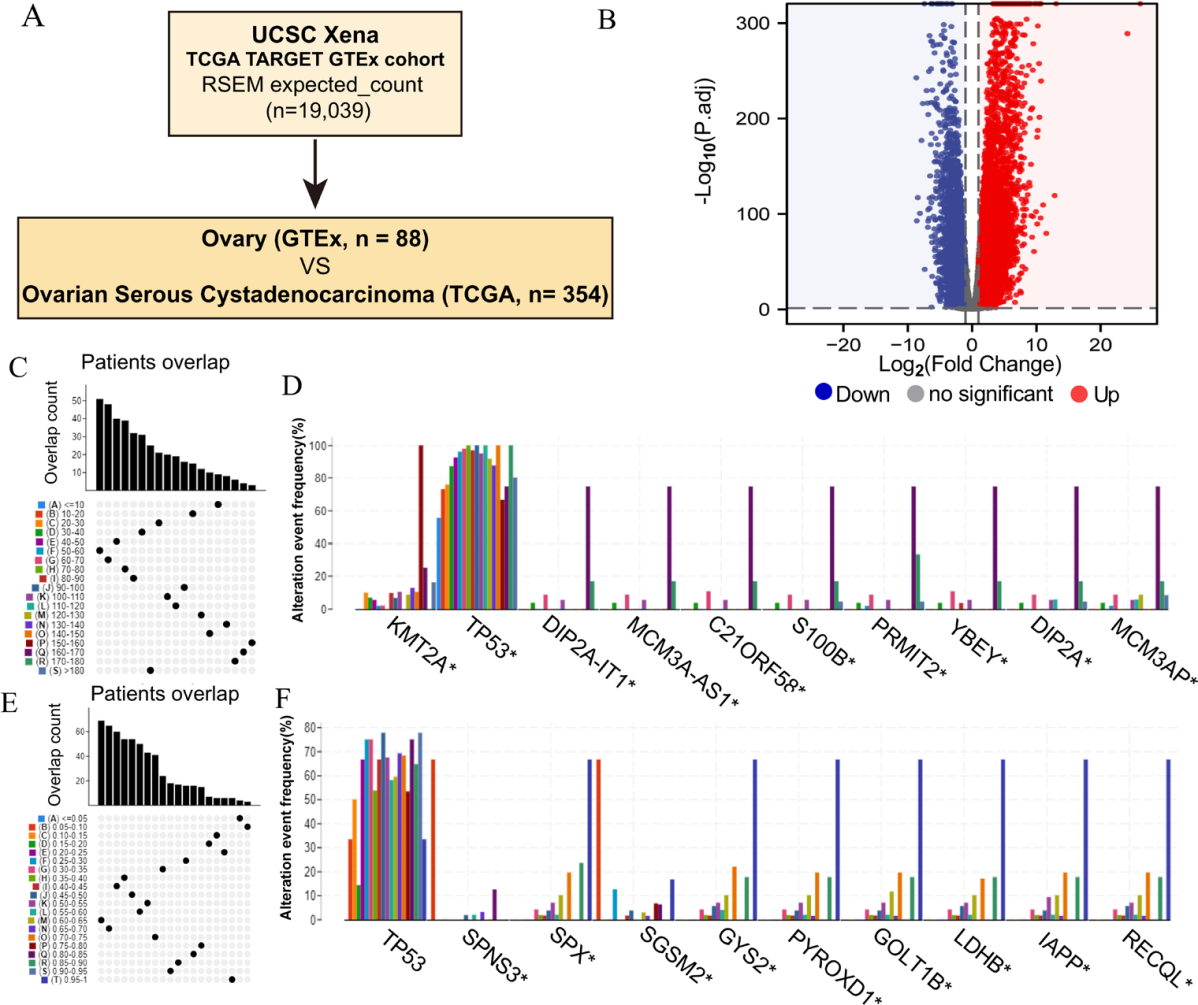
Identification of potential OV antigens. (A) The samples were collected from GTEx (*n* = 88) and TCGA (*n* = 354). (B) The Volcano Plot of gene expression. Genome segment reassortment (C, E) and the top mutation rate genes (D, F) in the two different datasets.

To identify potential mutation for OV, the samples of ovarian tissue (*n* = 88) and cancer tissue (*n* = 354) from TCGA and GTEx were extracted (Figure 2A). In our analysis, a total of 9651 differentially expressed genes were identified, with 6239 of these genes encoding tumor‐associated antigens (TAAs), as illustrated in Figure 2B. Additionally, we identified 12 812 mutated genes, which may potentially encode tumor‐specific antigens (TSAs), highlighting the genetic complexity and immunogenic potential within the context of OV and its association with ferroptosis‐related mutations (Figure 2C,E). Mutation analysis revealed that the TP53 gene was the most frequently mutated gene identified in our study. Moreover, our analysis revealed variations in both the total number of mutations and the frequency of genome changes among different genes. Specifically, genes such as KMT2A and DIP2A‐IT1 demonstrated a higher overall number of mutations, while genes like SPNS3, SPX, and SGSM2 exhibited a higher frequency of genome alterations, as depicted in Figure 2D,F. These observations offer significant insights into the genetic profile of OV, potentially aiding in the identification of key genes involved in driving tumorigenesis and influencing disease advancement.

**FIGURE 3:**
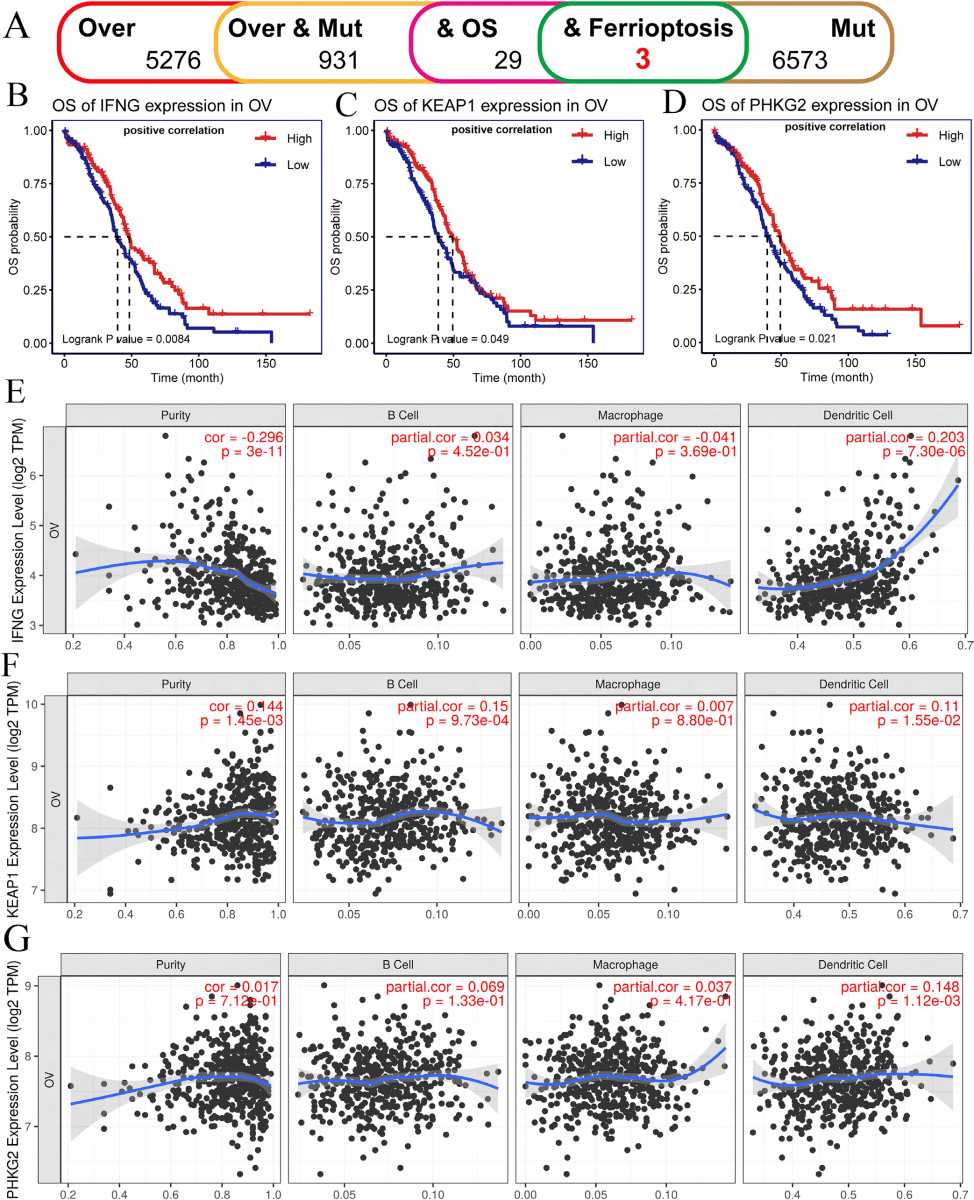
Identification of OV prognosis and APC infiltration‐related antigens. (A) The flow chart of screening. Kaplan–Meier curves showing the prognostic curves of IFNG, KEAP1, and PHKG2 genes in patients with OV tumors (B–D), high expression of these three genes indicates good prognosis. The correlation of IFNG, KEAP1, and PHKG2 gene expression levels with tumor purity, macrophages, dendritic cells, and B cell infiltration (E–G), *p* < 0.05, |cor| > 0.3 indicate a correlation. Mut, mutation; OS, overall survival; Over, overexpression.

Ferroptosis is one of the important types of cell death that is highly correlated with drug resistance and cancer development [27, 28]. In our investigation to elucidate the involvement and prevalence of FRGs, all known FRGs were incorporated into an analysis of overall survival. Among the 931 genes that exhibited both mutations and overexpression, three FRGs (IFNG, KEAP1, and PHKG2) were identified, as illustrated in Figure 3A. This discovery sheds light on the potential role of these FRGs in OV and their influence on patient outcomes. The elevated expression levels of IFNG, KEAP1, and PHKG2 were found to be correlated with a good prognosis, as depicted in Figure 3B–D. This association underscores the potential prognostic value of these FRGs in predicting disease progression and patient survival in OV cases with ferroptosis‐related mutations. We also analyzed the correlation of the expression of three FRGs with the infiltration of antigen‐presenting cells (B cell, macrophage, and dendritic cell) (Figure 3E–G). We found that the expression of the three FRGs did not affect the APC infiltration in TME.

**FIGURE 4:**
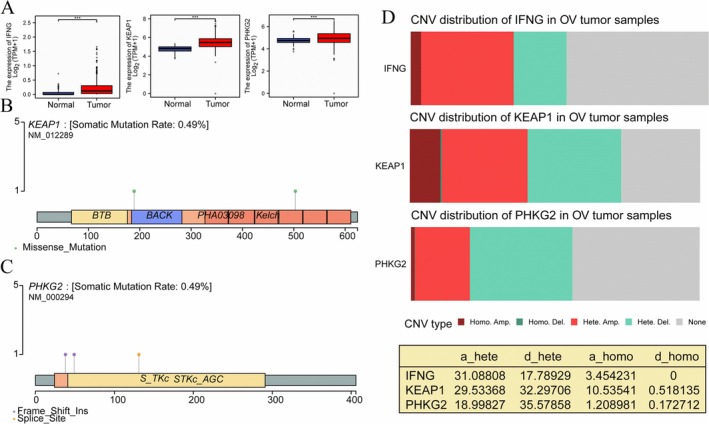
Genomic and transcriptomic analysis of potential target genes. (A) The expression of all three genes, including IFNG, KEAP1, and PHKG2 genes was higher in the tumor tissues, compared to those in the normal tissues. (B) The mutation sites of KEAP1 are located in specific regions within the gene sequence. (C) Similarly, mutation sites within PHKG2 are identified at distinct positions in the gene sequence. (D) Changes in gene copy numbers of IFNG, KEAP1, and PHKG2 genes are observed. *** indicates *p* < 0.001. CNV, copy number variant.

Furthermore, the overexpression of IFNG, KEAP1, and PHKG2 was also found in different data sets (Figure 4A). The mutations in IFNG, KEAP1, and PHKG2 were validated across various databases, as illustrated in Figure 4B,C. Additionally, copy number variants (CNVs) of these three genes were identified in OV samples, as depicted in Figure 4D. These findings suggest that IFNG, KEAP1, and PHKG2 could potentially serve as promising targets for the development of cancer vaccines aimed at enhancing the immune response against OV. Targeting these genes may enable the immune system to mount a more robust anti‐tumor response, presenting new therapeutic avenues for the treatment of OV.

### 3.2 Identification of Ferroptosis Molecular Subtypes Characterized by the Expression Patterns of IFNG, KEAP1, and PHKG2

**FIGURE 5:**
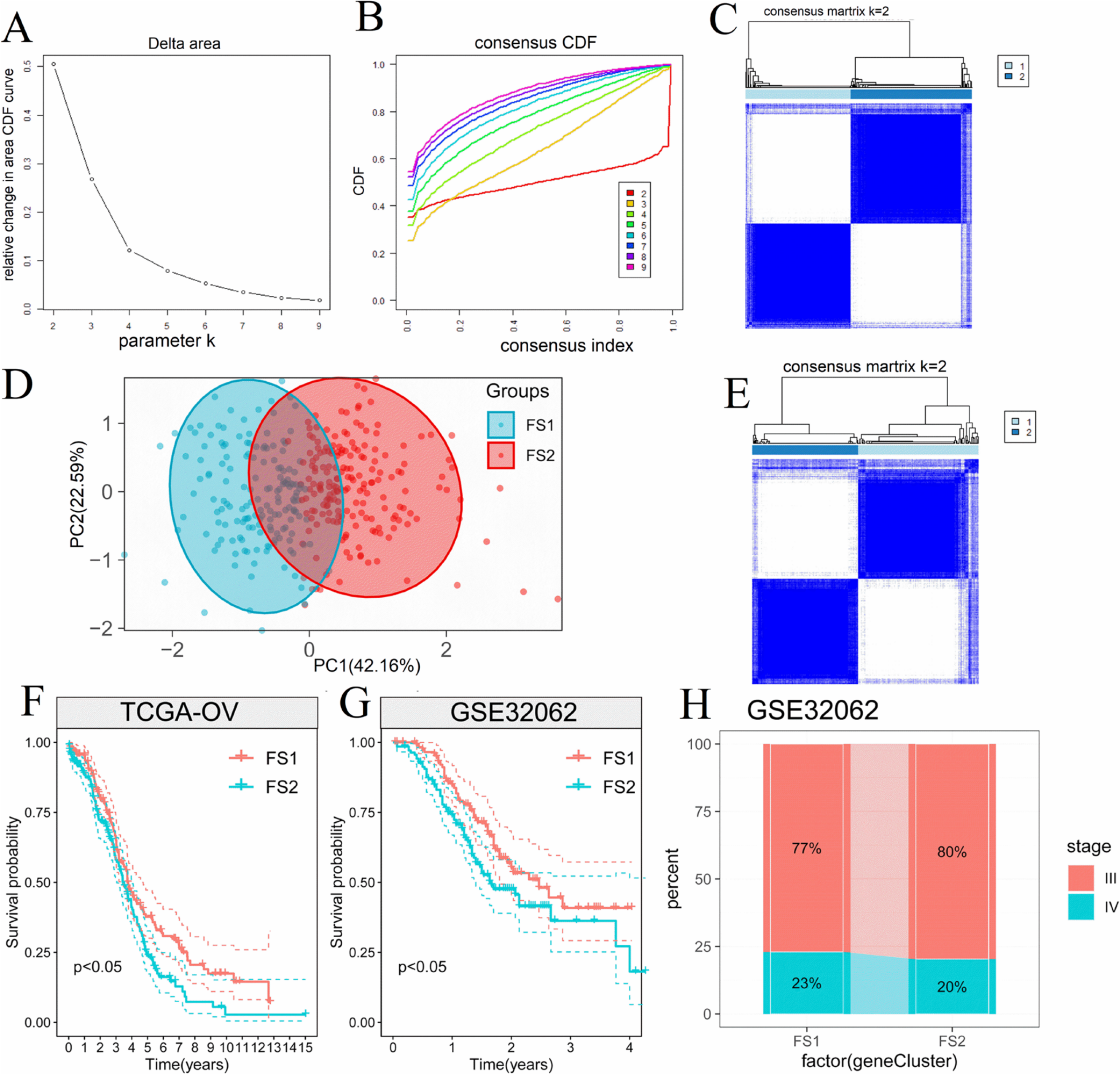
Identification of potential ferroptosis subtypes in OV patients. (A) Cumulative distribution function curve (B) δ area of ferroptosis‐related gene in the TCGA‐OV cohort. Clustering heatmap of TCGA‐OV cohort (C) and GEO cohort (D) (*k* = 2). (E) Principal component analysis of ferroptosis subtypes. Kaplan–Meier curve showing OS of TCGA‐OV (F) and GEO cohorts (G) of different ferroptosis subtypes. FS2 subtype was associated with a poorer prognosis in both the TCGA‐OV and GEO cohorts. (H) Distribution proportion of stage III and IV OV patients among different ferroptosis subtypes in GEO cohort. CDF, cumulative distribution function; FS1, ferroptosis subtypes 1; FS2, ferroptosis subtypes 2.

Ferroptosis has been proven to play an important role in OV cancer therapy [29]. FRGs were also found to influence cancer progression and immune cell infiltration [30, 31]. In this study, a total of 424 FRGs were utilized to construct consensus clusters. The TCGA dataset was employed to assess the expression profiles of these 424 FRGs. Through the analysis of cumulative distribution and functional triangle area, the parameter *k* = 2 was determined. Subsequently, two immune‐related genes were clustered (Figure 5A,B), and two distinct ferroptosis subtypes, FS1 and FS2, were identified (Figure 5C). Principal Component Analysis (PCA) further confirmed the differentiation between FS1 and FS2 in the GEO dataset (GSE32062), consistent with the TCGA‐OV analysis results (Figure 5D). GSE32062 was classified into two subtypes, FS1 and FS2 (Figure 5E). Importantly, the FS2 subtype was associated with a poorer prognosis in both the TCGA‐OV and GEO cohorts (Figure 5F), indicating its potential clinical relevance in predicting patient outcomes in OV cases with ferroptosis‐related mutations. Nevertheless, our analysis revealed that the distribution of the two ferroptosis subtypes showed no significant difference in clinical stages III and IV, as illustrated in Figure 5H. Overall, the findings from our study suggest that ferroptosis subtypes hold promise as predictive markers for clinical prognosis in OV, offering valuable insights into the potential implications of ferroptosis‐related mutations in disease progression and patient outcomes.

### 3.3 The Correlation Between Ferroptosis Subtypes and Mutation Burden

**FIGURE 6:**
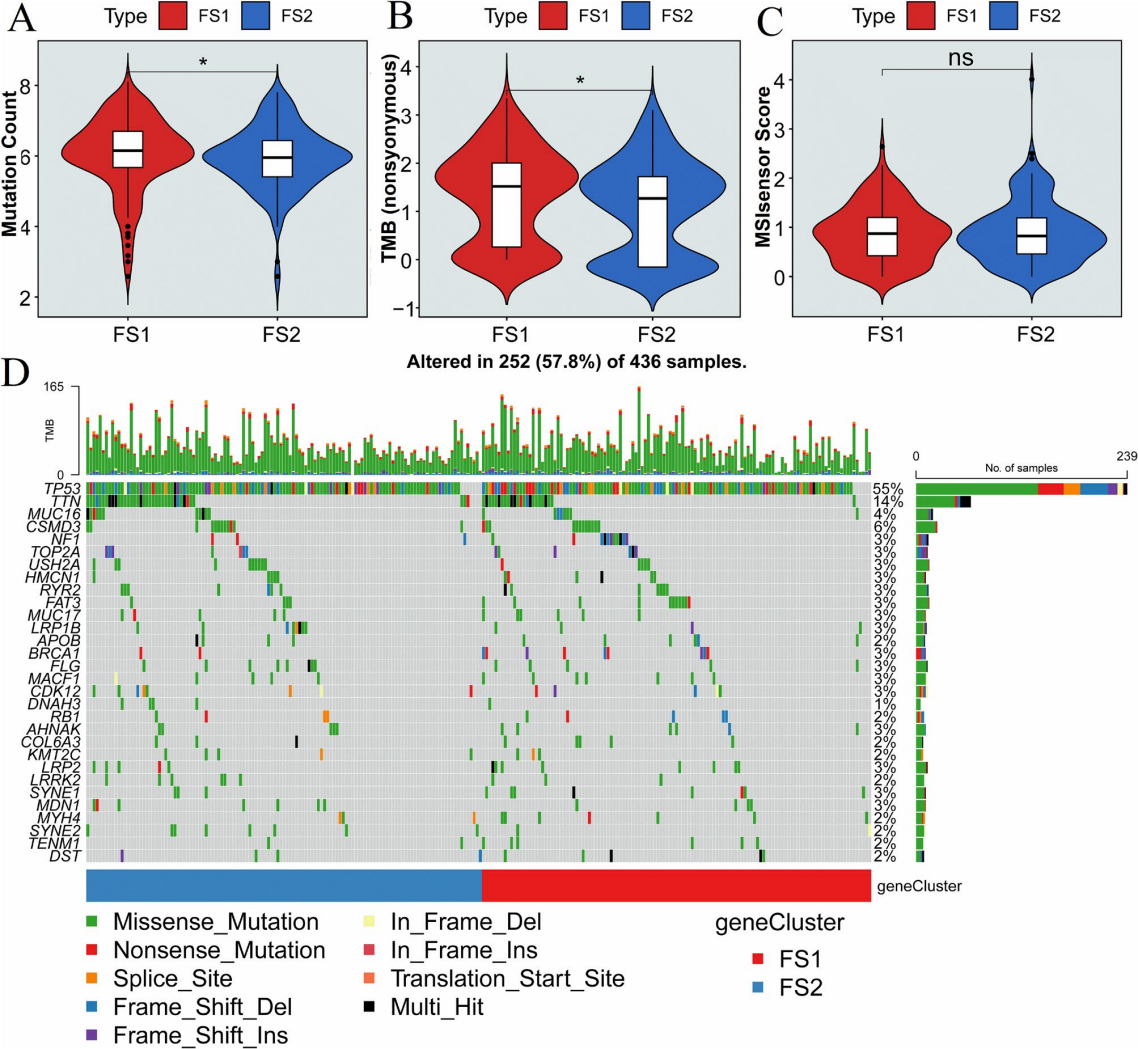
The correlation between TMB and ferroptosis subtypes. The mutation count (A), tumor mutation burden (B) and MSI sensor score (C) in different ferroptosis subtypes. (D) The waterfall chart shows mutation genes of different ferroptosis subtypes (FS1 and FS2). Mutation burden was correlated with the ferroptosis subtype. *p < 0.05, ns indicates no significance.

High TMB has been reported as a biomarker of response to immune therapy, and promotes antitumor immunity [32, 33]. The somatic mutation rate was also found to be associated with immunotherapy response and better prognosis [34, 35]. The cBioportal database was utilized to extract and analyze the TMB, microsatellite instability (MSI), and the number of mutations within the TCGA‐OV cohort. Our analysis revealed that the TMB and the number of mutations in the FS2 subtype were lower compared to those in FS1, as depicted in Figure 6A,B. These findings provide valuable insights into the mutational landscape associated with different ferroptosis subtypes in OV, suggesting potential differences in genomic instability and mutation burden between the identified subgroups. The MSI has no significant difference between FS1 and FS2 (Figure 6C). Top 30 genes, including TP53, TTN, MUC16, and CSMD3, showed different mutation burdens in FS1 and FS2 (Figure 6D). The data indicated that mutation burden was correlated with the ferroptosis subtype. The expression and mutation profiles of IFNG, KEAP1, and PHKG2 may serve as indicators of the tumor mutational burden (TMB) in OV, indicating their potential utility as predictive biomarkers for tumor response and prognosis. Furthermore, the identification of mutated FRGs in our study suggests their potential use as antigens for the development of cancer vaccines, presenting new avenues for immunotherapeutic strategies in the management of OV. These findings hold promise for advancing personalized treatment approaches and enhancing therapeutic outcomes in OV patients.

### 3.4 The Correlation of Ferroptosis Subtypes With CIP and ICD

**FIGURE 7:**
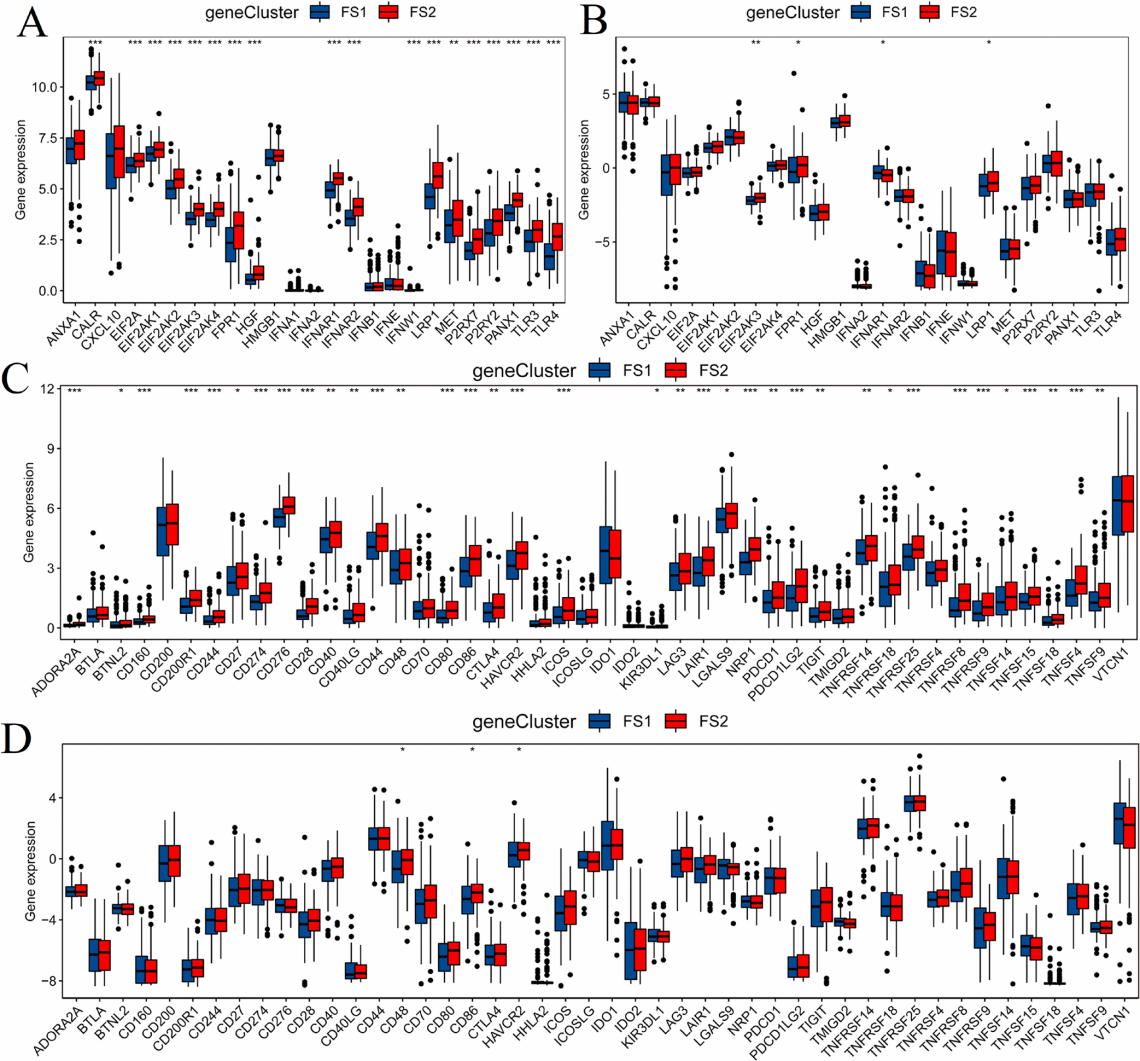
The correlation of ferroptosis subtypes with ICD and ICP. Twelve ICD‐related genes, including EIF2AK3, FPR1, IFNAR1, and LRP1were identified in TCGA‐OV (A) and GEO (B) cohorts. Thirty‐six and three ICP genes were identified in TCGA‐OV (C) and GEO (D) cohorts, respectively. *p < 0.05, **p < 0.01, ***p < 0.001, ****p < 0.0001.

Targeting immune checkpoint proteins (ICPs) is one of the most promising strategies for cancer therapy, and drugs targeting ICP have been approved by the FDA [36, 37]. Immunogenic cell death (ICD) is usually induced by radiotherapy, chemotherapy, and oncolytic virus therapy, and mediated by damage‐associated molecular patterns (DAMPs) [38]. To clarify the correlation of ferroptosis subtypes with CIP and ICD, we evaluated the distinct expressions of immune checkpoint (ICP) and ICD modulators in the two ferroptosis subtypes of OV. By analyzing the expression levels of these key regulators, we aimed to gain insights into the immune microenvironment and cell death mechanisms associated with each ferroptosis subtype. 25 ICD‐related genes were included in this study, and 18 of those genes were differentially expressed in FS1 and FS2 from the TCGA‐OV cohort. To further validate our findings, we utilized the Gene Expression Omnibus (GEO) database in our analysis. By corroborating our results with data from an independent dataset, we aimed to strengthen the robustness and reliability of our conclusions regarding the association between ferroptosis subtypes, immune modulation, and OV outcomes. We finally identified 12 genes, including EIF2AK3, FPR1, IFNAR1, and LRP1, in the two databases (Figure 7A,B). In our investigation, a total of 46 ICPs were analyzed using data from the TCGA‐OV and GEO databases. Among these, 36 genes were identified in the TCGA‐OV cohort, while three genes were identified in the GEO cohort, as presented in Figure 7C,D. Our findings suggested that the ICPs may not be effective in distinguishing between the FS1 and FS2 ferroptosis subtypes. However, we discovered that CD48, CD86, and HAVCR2 were common factors in both cohorts, indicating a potential synergistic effect by targeting both ferroptosis pathways and immune checkpoints in cancer therapy. This integrated approach could hold promise for enhancing therapeutic strategies in the treatment of OV.

### 3.5 Cellular and Molecular Features Characterized by the Expression Patterns and Mutation Profiles of IFNG, KEAP1, and PHKG2

**FIGURE 8:**
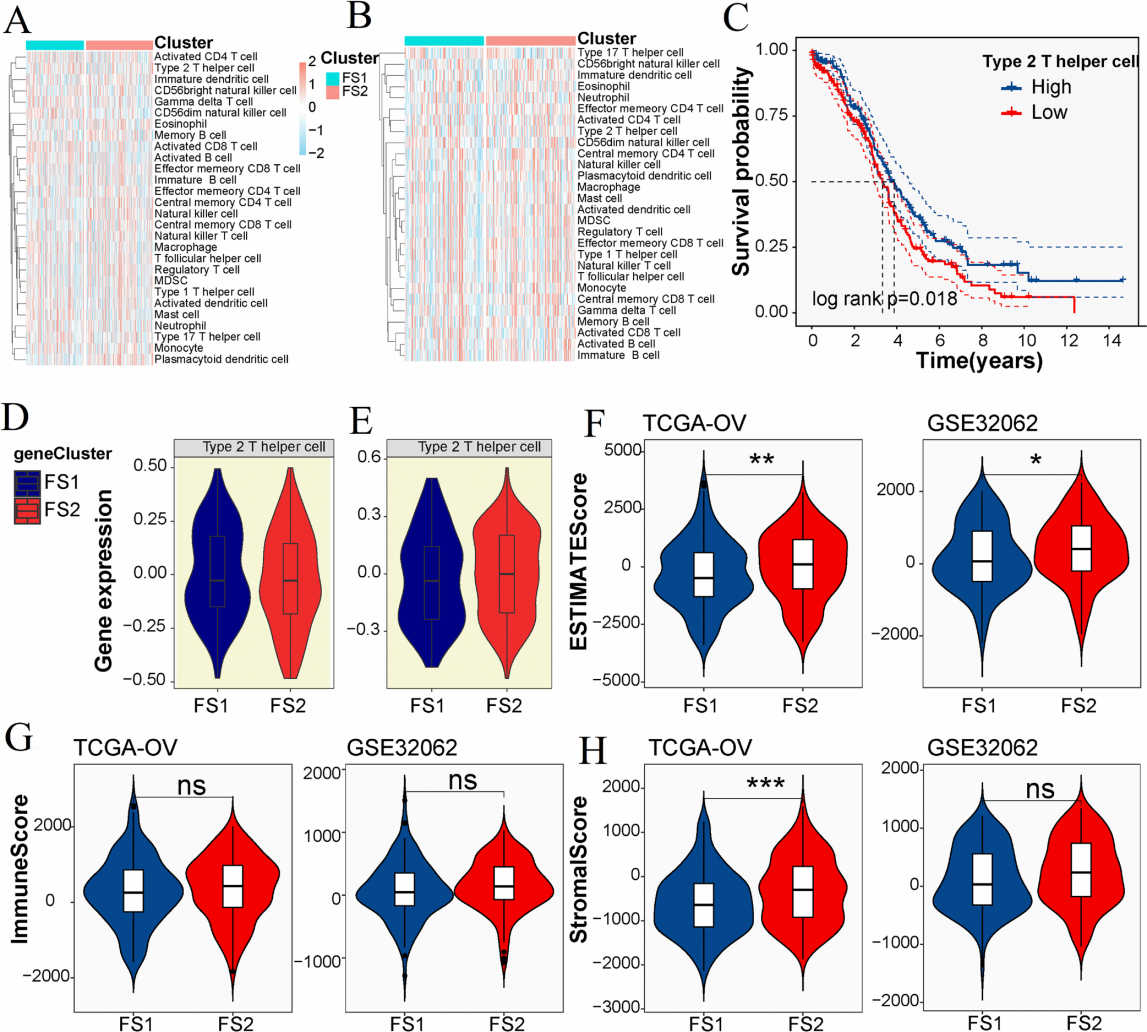
Cellular and molecular characteristics of ferroptosis subtypes. Heat map of enrichment scores for 28 immune cell markers in TCGA‐OV (A) and GEO (B) cohorts. (C) The survival curve of Type 2 T helper cell high and low groups (*p* = 0.018). Gene expression of Type 2 T helper cells in FS1 and FS2 subtypes in TCGA‐OV (D) and GEO (E) cohorts. The ESTIMATE score (F), immune score (G) and matrix score (H) of FS1 and FS2 subtypes in TCGA‐OV and GEO cohorts. *p < 0.05, **p < 0.01, ***p < 0.001.

Cancer therapy is largely dependent on the immune status of TME [39]. To get better performance of cancer diagnosis and treatment, the tumor's immune status should be clarified. We evaluated the immune status in TME by scoring the 28 genes identified in the TCGA‐OV and GSE32062 cohorts, and the immune cell components were divided into two clusters (Figure 8A,B). The Kaplan–Meier curve analysis demonstrated that the level of type 2 helper T cells among the 28 immune signatures could effectively differentiate between high and low subjective survival probabilities, as depicted in Figure 8C. However, no significant difference in the level of type 2 helper T cells was observed between the FS1 and FS2 subtypes, as shown in Figure 8D,E. By calculating the ESTIMATE, immune, and stroma scores using the ESTIMATE database, we observed a higher ESTIMATE score in FS2 compared to FS1 (Figure 8F), while the immune score showed no significant difference between the two subtypes (Figure 8G). These results were consistent across the TCGA‐OV and GEO cohorts. Furthermore, the stroma score was found to be higher in FS2 than in FS1 in the TCGA‐OV cohort, although no significant difference was observed in the stroma score between FS1 and FS2 in the GEO cohort. The immune score could serve as a valuable indicator for assessing T‐lymphocyte abundance in OV tissue. Our study highlights the potential of IFNG, KEAP1, and PHKG2 as predictive markers for T cell infiltration and the tumor microenvironment status in OV. The expression and mutation profiles of these genes offer insights into the immune response within the tumor microenvironment, providing valuable information on the interplay between the tumor and the immune system.

### 3.6 The Mutational and Expression Patterns of KEAP1, and PHKG2 Exhibit a Strong Association With the Genomic Mutation Status of Cancer Cells

**FIGURE 9:**
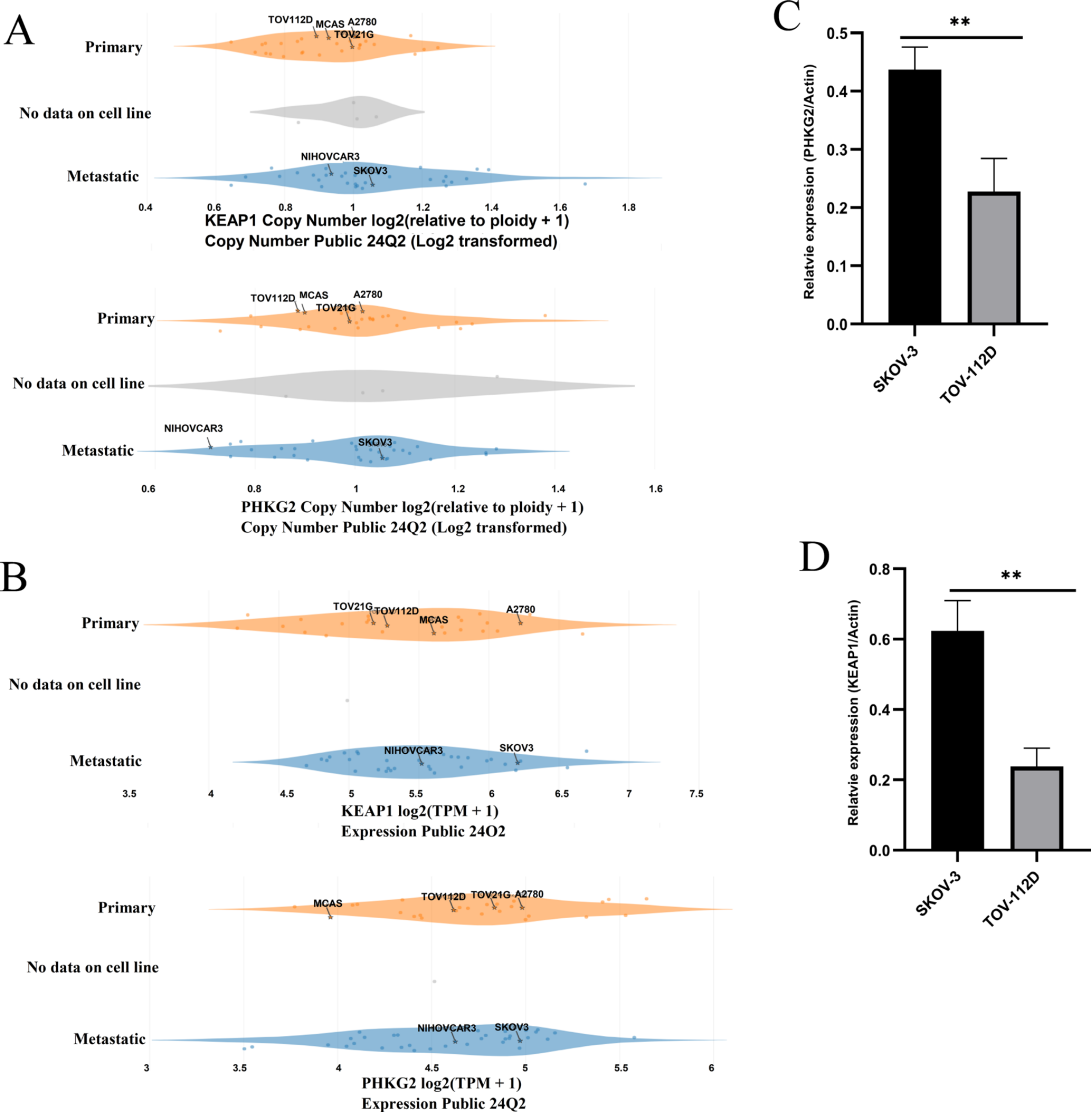
The association between mutational and expression patterns of KEAP1, and PHKG2 and the genomic mutation status of cancer cells. (A) Expression levels of PHKG2 and KEAP1 across six ovarian cancer cell lines. (B) Copy number variations of PHKG2 and KEAP1 in six ovarian cancer cell lines. (C) Relative expression of KEAP1 in TOV‐112D and SKOV‐3 cell lines. (D) Relative expression of PHKG2 in TOV‐112D and SKOV‐3 cell lines. **p < 0.01.

The TMB is recognized as a predictor of immunotherapy response. To further validate the predictive value of mutations and expression levels of IFNG, KEAP1, and PHKG2 in determining TMB, we utilized the Dependency Map (DepMap) portal. Data from six OV cell lines (A2780, TOV‐112D, TOV‐21G, MCAS, NIHOVCAR3, and SKOV‐3) with varying mutation statuses were collected [40]. Analysis of damaging mutations, hotspot mutations, gene expression, and copy number variations of the three genes was conducted across the selected cell lines. Our findings indicate that the expression and copy number of PHKG2 and KEAP1 are correlated with the mutation burden in these cell lines (Figure 9A,B). However, our investigation did not detect any discrepancies in damaging mutations, hotspot mutations, gene expression, and copy number variations of IFNG among the six cell lines (data not shown). Differential expression of KEAP1 and PHKG2 in TOV‐112D (low mutation) and SKOV‐3 (high mutation) was confirmed through RT‐qPCR (Figure 9C,D). The roles of KEAP1 and PHKG2 in maintaining genomic stability and the potential significance of targeting these genes for OV therapy warrant further investigation.

## 4 Discussion

Ferroptosis has been implicated in a multitude of physiological and pathological processes, with a particular focus on its role in cancer progression and metastasis [12]. Numerous FRGs have been identified and confirmed across various datasets. The prognostic relevance of these FRGs has been explored in cancer patients using a variety of bioinformatics methodologies. This comprehensive exploration of FRGs and their prognostic implications highlights their potential as valuable biomarkers for predicting outcomes and guiding treatment decisions in cancer patients. The utilization of bioinformatics tools has been instrumental in uncovering the prognostic value of FRGs, enhancing our understanding of their role in cancer progression and prognosis [41, 42]. Prognostic genes differ across tumor types, highlighting the heterogeneity of FRGs [43]. In our current study, we assessed and compared FRGs in different ferroptosis subtypes. We found that the expression of FRGs varied among ferroptosis subtypes. Notably, as previously reported, “hot” tumors, characterized by increased immune activity, often exhibit favorable responses to immunotherapy [44]. In our study, we delved into the molecular signatures of genes linked to distinct ferroptosis subtypes and noted that FS1, characterized by a low mutation burden, aligned with “cold” tumors, whereas FS2, marked by a high mutation burden, aligned with “hot” tumors. Accurately distinguishing between these ferroptosis subtypes is essential for tailoring precise and effective treatment strategies for OV. This classification based on mutation burden and molecular characteristics provides valuable insights that can guide personalized approaches to treatment in OV, emphasizing the importance of recognizing and targeting specific ferroptosis subtypes for improved patient outcomes.

TMB was correlated with tumor microenvironment status [45]. Low TMB in OV may limit the utility of immune checkpoint blockade and vaccines in cancer therapy [46, 47]. The TMB serves as a critical factor in cancer biology, influencing immune response, tumor progression, and treatment outcomes. The previous study found a novel association of TP53 with CD8+ T cell infiltration in the TME in patients with different TMB and showed that TP53 status could act as a prognostic marker [48]. These results indicated the importance of TMB as a potential therapeutic target and biomarker in cancer treatment. In our present study, the correlation between TMB and FRGs was analyzed. Our data revealed significant variations in the TMB among different ferroptosis subtypes. Specifically, FS2 patients with elevated TMB levels may benefit from immune‐based therapies. Vaccines have long been employed for the prevention of pathogen infections [49]. Tumor‐associated antigen (TAA) and TSA has been studied and remarkable progression have been achieved [50]. The TAA or TSA‐based vaccines are already undergoing preclinical and clinical trials [51]. The high level of TMB provides a neoantigen library for cancer vaccine development. Clarifying the correlation of ferroptosis and TMB contributes to the development of OV cancer treatment strategies targeting ferroptosis and neoantigens.

Both passive and active immunotherapy have made huge achievements in cancer therapy. Antibodies targeting immune checkpoints, such as PD1, PDL1, and LAG3, have been approved by FDA for cancer therapy [52, 53]. Inconsistent with passive immunotherapy mechanisms, active immunotherapy relies not only on T cell infiltration but also on antigen‐presenting cells and help T cells [54]. Our analysis revealed that patients with high TMB levels in the FS2 ferroptosis subtype of OV exhibit increased infiltration of Th2 and antigen‐presenting cells (APCs) compared to those in the FS1 subtype. This suggests that FS2 patients may potentially benefit from a combination of active immunotherapy, which involves stimulating the immune system to target cancer cells, with passive immunotherapy, which utilizes external agents to enhance the immune response.

The identification of IFNG, KEAP1, and PHKG2 in our present study underscores the importance of more detailed patient classification for advancing precision medicine in OV treatment. High expression levels of these genes have been associated with better prognosis and enhanced responses to immunotherapy, highlighting their potential as valuable biomarkers for patient stratification. Compared to individual gene markers, utilizing combinations of multiple genes offers a more comprehensive approach to formulating tailored treatment strategies for patients. It is imperative to conduct in‐depth investigations into the functional roles of KEAP1 and PHKG2 in regulating genomic stability in OV cells. Understanding the contribution of these genes to mutational processes and tumor evolution may unveil their potential as promising therapeutic targets. By elucidating the mechanisms through which targeting IFNG, KEAP1, and PHKG2 could influence OV progression and treatment response, we identified three FRGs associated with OV prognosis, and observed that only the expression and copy number of KEAP1 and PHKG2 were correlated with TMB. IFNG, functioning as an immune response marker, is not directly involved in tumor mutagenesis but rather a consequence of the immune response triggered by mutations.

TCGA data is a pivotal resource for studying the complex interactions within the TME and cancer as well as cancer cells. Previous studies have reported that the infiltration of macrophages was correlated with poor prognosis of OV patients using the TCGA database [55]. Our present study found that high TMB was associated with increased T cell infiltration and exhibited a distinct gene signature using TCGA data.

Consequently, our analysis provides evidence that IFNG, KEAP1, and PHKG2 act as critical players in OV patients to regulate the T cell infiltration into the TME and have a significant clinical impact on the patients' survival. Tumor‐associated macrophages (TAMs) are an important component of TME and are associated with immunosuppression and poor prognosis [56]. IFNG was reported to prevent the generation of TAMs [57]; KEAP1 was confirmed to affect the recruitment and differential polarization of immunosuppressive macrophages [58]. Further research needs to verify the complex interactions between the three key genes and TAMs in OV development.

Our study faces certain limitations that should be acknowledged. Firstly, our study primarily focused on elucidating the association between three novel FRGs and OV prognosis. While our findings provide valuable insights into this aspect, they are based on observational data, which may not fully capture the function and complexity of these key genes in clinical and in vivo settings. Further studies, including in vivo animal models and clinical investigations, are warranted to validate our findings. Additionally, although we investigated the link between three key genes and the genomic mutation status of cancer cells, our present study did not extensively explore the roles and mechanisms of these interactions. Further research is warranted to validate these findings and explore their clinical implications in OV treatment.

In conclusion, our study has identified specific gene combinations that can aid in the selection of patients who are likely to benefit from immunotherapy, particularly in the context of TMB. By leveraging these gene signatures, clinicians can optimize treatment decisions and improve outcomes for OV patients undergoing immunotherapy.

## Author Contributions

**Leilei Cao:** formal analysis, writing – original draft, writing – review and editing, investigation, and methodology. **Yiqin Ouyang:** formal analysis, investigation, methodology, writing – original draft, conceptualization, and supervision. **Wei Lu:** formal analysis, investigation, and writing – original draft. **Xiao Qi:** formal analysis, investigation, writing – review and editing, and methodology. **Zhijie Wang:** conceptualization, supervision, investigation, writing – review and editing. **Jingshuai Wang:** conceptualization, supervision, investigation, writing – review and editing.

## Conflicts of Interest

The authors declare no conflicts of interest.

## Data Availability

The data that support the findings of this study are openly available in GSE32062 at https://xenabrowser.net/datapages/.
